# Structural insight into GPR55 ligand recognition and G-protein coupling

**DOI:** 10.1038/s41422-024-01044-w

**Published:** 2024-10-31

**Authors:** Ruixue Xia, Qingning Yuan, Na Wang, Li Hou, Junpei Abe, Jing Song, Yukishige Ito, H. Eric Xu, Yuanzheng He

**Affiliations:** 1 Laboratory of Receptor Structure and Signaling, HIT Center for Life Sciences, School of Life Science and Technology, Harbin Institute of Technology, Harbin, Heilongjiang China; 2 State Key Laboratory of Drug Research, Shanghai Institute of Materia Medica, Chinese Academy of Sciences, Shanghai, China; 3 Shanghai Advanced Electron Microscope Center, Shanghai Institute of Materia Medica, Chinese Academy of Sciences, Shanghai, China; 4 Graduate School of Science, Osaka University, Toyonaka, Osaka Japan; 5 Osaka International Cancer Institute, Osaka, Osaka Japan; 6 School of Life Science and Technology, ShanghaiTech University, Shanghai, China; 7 Frontiers Science Center for Matter Behave in Space Environment, Harbin Institute of Technology, Harbin, Heilongjiang China

**Keywords:** Cryoelectron microscopy, Hormone receptors

Dear Editor,

GPR55 was originally considered the third cannabinoid receptor (CB3) due to its high expression in the central nervous system and its association with endocannabinoids, such as *N*-arachidonoylethanolamine (AEA).^1^ However, subsequent studies indicate that GPR55 lacks a traditional ‘cannabinoid-binding pocket’,^2^ and the activation of GPR55 by endocannabinoids may be mediated by other receptors. In 2007, Oka et al. demonstrated that L-α- lysophosphatidylinositol (LPI) robustly activates GPR55 and that this effect is absent in GPR55 knockdown cells.^3^ Following similar discoveries by other groups, LPI has gradually been accepted as the endogenous ligand of GPR55. In the meantime, synthetic CB1 antagonists, such as AM251 and SR141716A, have been shown to activate GPR55 in cell-based reporter assays.^4^ Despite the lasting debate over whether GPR55 is a cannabinoid receptor, it is known to play crucial roles in the nervous and metabolic systems. In the nervous system, GPR55 is highly expressed in hippocampus, brain stem, cerebellum, frontal cortex, hypothalamus, and striatum. It is closely associated with sensation, cognition, and pain perception, making it a promising target for treating Parkinson’s disease.^5^ Of note, GPR55 agonists, O-1602 and palmitoylethanolamide, have been used to relieve pain in animal models. In the metabolic system, GPR55 is abundantly expressed in adipocytes, pancreas islet, gastrointestinal tract and adrenals. Consistent with its expression pattern, GPR55 regulates insulin secretion, intestinal inflammatory factors, and plasma glucose levels, presenting a promising target for metabolic disorders, such as diabetes and nonalcoholic steatohepatitis.^6^ Notably, GPR55 agonists AM251 and SR141716A have been demonstrated to reduce body weight by decreasing food intake in animal models.^1^ Given the significance of GPR55 in both nervous and metabolic systems, we aimed to gain structural insight into GPR55 ligand recognition and G-protein coupling.

A variety of small molecules, lipids, and even peptides have been implicated as ligands of GPR55. We first sought to identify a potent activator of GPR55. While GPR55 primarily signals through G_13_ pathway, it has been reported to induce G_q_ signaling under certain circumstances. We examined several well-documented GPR55 ‘binders’, including AM251, LPI, O-1602, oleylethanolamide, palmitoylethanolamide, PACAP27 peptide and SQ-LPG,^7^ using a bioluminescence resonance energy transfer (BRET) dissociation assay of G_13_. Results showed that LPI most robustly activated GPR55 with an EC_50_ in the micromolar range (~0.34 µM) (Supplementary information, Fig. S1a and Table S1), consistent with the previous report.^3^ In contrast, O-1602, AM251 and SQ-LPG only weakly activated GPR55, and all other molecules failed to activate GPR55 in the BRET dissociation assay of G_13_. We then examined the activities of LPI, AM251 and SQ-LPG in a BRET assay of G_q_ dissociation. Results indicated that only LPI activated G_q_ signaling, but to a much lesser extent than G_13_ signaling (Supplementary information, Fig. S1b), consistent with previous observations that GPR55 mainly signals through G_13_ signaling.^1^ Similarly, in the NanoBiT recruitment assay of β-arrestin 1, only LPI was able to recruit β-arrestin 1 to GPR55 (Supplementary information, Fig. S1c). We also tested whether endogenous cannabinoid ligands AEA and 2-arachidonoylglycerol (2-AG) could activate GPR55. In the BRET dissociation assay of G_13_, neither AEA nor 2-AG exhibited any discernible activity (Supplementary information, Fig. S1f). We further tested whether the synthetic cannabinoid CP55940 was capable of activating GPR55. Interestingly, instead of activation, CP55940 exhibited inhibitory activity on GPR55 in the BRET assay, suggesting that CP55940 might act as an inverse agonist of GPR55 (Supplementary information, Fig. S1f). Collectively, these data support the conclusion that LPI is an authentic ligand of GPR55.

Structural determination of GPR55 is challenging as the wild-type (WT) GPR55 is not stable. To overcome the hurdle, we fused GPR55 with GFP at its C-terminus and screened mutants of GPR55 using fluorescence-detection size-exclusion chromatography (FSEC).^8^ A C287^7.52^V mutant (superscripts refer to the Ballesteros-Weinstein numbering) stood out as it significantly reduced the aggregation peak of GPR55 in the FSEC analysis (Supplementary information, Fig. S1d), while displaying a similar or slightly improved response to LPI in the BRET dissociation assay of G_13_ (Supplementary information, Fig. S1e). We therefore used the C287^7.52^V mutant as the backbone of GPR55 for structural determination (Supplementary information, Fig. S2a). Since GPR55 primarily signals through G_13_, we aimed to obtain a GPR55/G_13_ complex. To facilitate complex assembly, we employed a NanoBiT tethering strategy, which had proven effective in numerous complexes with weak or dynamic associations.^9^ We fused the C-terminus of GPR55 with the large fragment of NanoBiT (LgBiT) and the C-terminus of Gβ with the innovative high-affinity small fragment of NanoBiT (HiBiT). A miniGα_13_ was used for the complex assembling (see Supplementary information, Materials and Methods). The use of mini G-proteins facilitates the structural determination of G-protein-coupled receptor (GPCR)/G-protein complexes, and numerous studies have demonstrated that the G-protein-engaging conformations obtained from mini G-proteins align closely with those of regular G proteins. Therefore, for convenience, we designate GPR55/miniG_13_ as GPR55/G_13_. We expressed the receptor and the G-protein in sf9 cells. The purified complexes exhibited well-separated peaks in the size-exclusion chromatography profile (Supplementary information, Fig. S2b).

We solved the structures of LPI (16:0)- and AM251-bound GPR55 in complex with G_13_ by single particle analysis of cryo-electron microscopy (cryo-EM) at resolutions of 2.85 Å and 3.19 Å, respectively (Supplementary information, Fig. S3 and Table S2). The structures exhibit the typical GPCR/G-protein complex conformation in which the G-protein engages with the receptor mainly through its Gα subunit (Fig. 1a, b). The LPI-bound GPR55/G_13_ complex shows a better density map than the AM251-bound GPR55/G_13_ complex, which may be due to the higher affinity of LPI than the weak agonist AM251. The receptor side is well resolved except the N-terminus (1–9 aa) and C-terminus (297–319 aa), due to high flexibility of these regions. The receptor structures of LPI-bound GPR55 and AM251-bound GPR55 are nearly identical with a root mean square deviation (RMSD) of 0.473 Å, showing only minor changes in the extracellular loop 2 (ECL2) (Supplementary information, Fig. S4a).

**Fig. 1:**
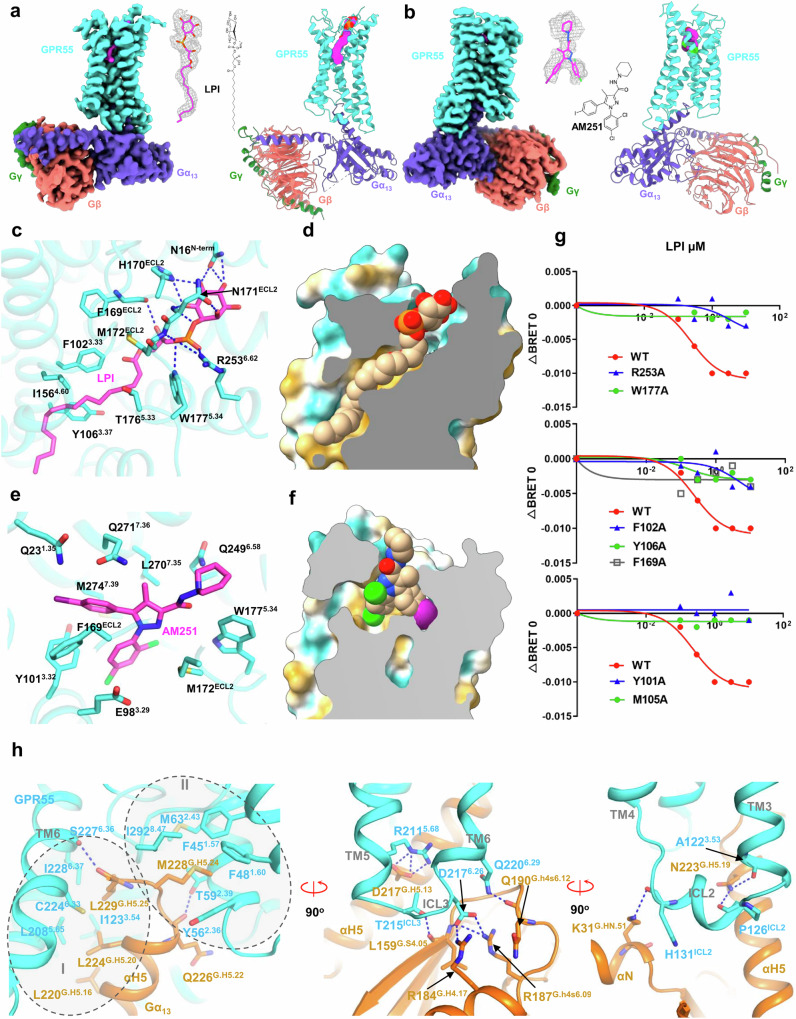
Structural insights into ligand recognition and G-protein coupling of GPR55. **a**, **b** Overall structures of LPI-bound and AM251-bound GPR55/G_13_ complexes, respectively. Left panel, orthogonal view of the cryo-EM density map; right panel, model of the complex in the same view and color scheme with the left panel. **c** LPI binding and recognition mode in GPR55. **d** Hydrophobicity analysis of the LPI-binding pocket in GPR55. **e** AM251 binding and recognition mode in GPR55. **f** AM251 only occupies the middle-upper part of the hydrophobic pocket of GPR55. **g** BRET assays of G_13_ dissociation for GPR55 ligand-binding pocket mutants. Data are presented as means ± SD; *n* = 3. **h** Details of the G_13_ engagement with GPR55.

LPI is well-resolved in the orthosteric pocket. The inositol head and phosphate group make extensive polar interactions with the upper part of pocket (Fig. 1c; Supplementary information, Fig. S4b), while the acyl tail snuggles into a highly hydrophobic groove in the lower part of pocket (Fig. 1d). Specifically, N16^N-term^, H170^ECL2^ and N171^ECL2^ form a hydrogen-bond network with LPI’s inositol ring; M172^ECL2^, W177^5.34^, and R253^6.62^ form polar interactions with LPI’s phosphate group, clamping LPI in position for receptor binding. The backbone carbonyl group of F169^ECL2^ forms a hydrogen-bond interaction with the sn-1 hydroxyl group of the glycerol; and F102^3.33^, I156^4.60^ and Y106^3.37^ shape the hydrophobic tunnel for the acyl tail. The tunnel is directly connected to an opening formed by TM3, TM4 and TM5 in the middle of the bilipid plasma membrane (Supplementary information, Fig. S4d), which serves as a lateral entry for lysolipids in the membrane, similar to what we observed in GPR3 and GPR174.

In contrast, AM251 occupies only the upper portion of the ligand-binding pocket (Fig. 1f), surrounded by hydrophobic residues such as W177^5.34^, M172^ECL2^, F169^ECL2^, Y101^3.32^, M274^7.39^, and L270^7.35^ within the core of the pocket. Several hydrophilic residues, including E98^3.29^, Q23^1.35^, Q271^7.36^ and Q249^6.58^, are positioned on the pocket’s periphery (Fig. 1e). The binding of AM251 to the receptor is primarily mediated by hydrophobic interactions (Supplementary information, Fig. S4c). A superimposition of AM251-bound GPR55 with LPI-bound GPR55 shows that the binding pose of AM251 aligns well with that of LPI. The aminopiperidine group of AM251 makes close contact with ECL2, in a similar way as the inositol group of LPI does. Akin to the phosphate-glycerol stem of LPI, the pyrazole-3-carboxamide group of AM251 makes key contact with F169^ECL2^, M172^ECL2^, F246^6.55^ and L270^7.35^ (Supplementary information, Fig. S4e). To further assess the stability of AM251 binding in GPR55, molecular dynamics (MD) simulations were performed. A 200-ns triplicated run confirmed that the binding pose of AM251 remains stable in GPR55 (Supplementary information, Fig. S4f and Video S1).

We utilized BRET assays to validate the structural observations of ligand binding in GPR55. The residues W177^5.34^ and R253^6.62^ made key interactions with the phosphate group of LPI. Mutating these residues to alanine completely abolished the response to LPI (Fig. 1g), highlighting their essential role in LPI recognition. Similarly, F169^ECL2^A mutation largely decreased GPR55’s response to LPI in the BRET assay, consistent with its crucial role in interacting with the sn-1 hydroxyl group. Y106^3.37^, F102^3.33^, Y101^3.32^ and M105^3.36^ shape the hydrophobic pocket; the mutations of these residues dramatically impaired receptor activation. Interestingly, although N16^N-term^, H170^ECL2^ and N171^ECL2^ made extensive interactions with the inositol ring, mutations of these residues only slightly decreased pEC_50_ (Supplementary information, Fig. S5a and Table S3) and unexpectedly increased the Emax (Supplementary information, Fig. S5b), suggesting that the inositol ring binding may increase affinity but is not essential for receptor activation. Additionally, the M172^ECL2^A mutation had little effect on receptor activity, possibly due to the weak and redundant binding to the phosphate group (Fig. 1c; Supplementary information, Figs. S4b, S5b). All mutants used were expressed at similar levels, as confirmed by a surface expression assay (Supplementary information, Fig. S5c).

We compared the binding mode of LPI in GPR55 with other lysophosphoplipids, including lysophosphatidylserine (LysoPS) in GPR174, lysophosphatidic acid (LPA) in LPAR1, sphingosine-1-phosphate (S1P) in S1PR1 and lysophosphatidylcholine (LPC) in GPR119. Except LysoPS, the phosphate heads of all other lysophosphoplipids stand upright to the extracellular side, however, their binding poses differ significantly (Supplementary information, Fig. S6a). We then compared LPI in GPR55 with its closest analog, LPC in GPR119, in details. In contrast to the extensive polar interactions of LPI with GPR55, LPC only loosely associates with GPR119 through polar interactions mediated by Q65^2.64^ and R262^7.36^ (Supplementary information, Fig. S6b).

A central question regarding GPR55 is whether it is a cannabinoid receptor. We compared the binding mode of AM251 in GPR55 with various CB1 binders in CB1, including agonists AMG615^10^ and AM841,^11^ as well as the antagonist taranabant.^12^ All these CB1 binders show similar binding patterns largely different from the AM251-engaging mode in GPR55 (Supplementary information, Fig. S6c). A close comparison between AM251 in GPR55 and AM841 in CB1 reveals that W177^5.34^ plays a crucial role in AM251 binding to GPR55, while Y275^5.39^ and H178^2.65^ anchor AM841 for binding to CB1 (Supplementary information, Fig. S6d). These observations indicate that the ligand binding mode of GPR55 is entirely distinct from that of the cannabinoid receptor.

It is intriguing that AM251, an antagonist of CB1, acts as an agonist for GPR55. To explore the underlying mechanism, we first docked AM251 into the structure of taranabant-bound CB1.^12^ Given that AM251 shares a similar backbone with taranabant, it was easily accommodated into the taranabant-bound CB1 (Supplementary information, Fig. S6e). Structural comparison of AM251-bound GPR55 with AM251-docked CB1 revealed that AM251 adopts distinct binding modes within the ligand-binding pockets of GPR55 and CB1. In CB1, the aminopiperidine group of AM251 pushes TM1 and TM2 outward (Supplementary information, Fig. S6f), maintaining the receptor in an inactive conformation, as the inward movement of TM1 and TM2 is crucial for ligand-induced activation of CB1.^13^ In contrast, in GPR55, the aminopiperidine group faces the extracellular side, interacting with ECL2 and W177^5.34^; both interactions are essential for LPI-induced GPR55 activation (Fig. 1c, d, g). Additionally, F169^ECL2^ forms a π–π interaction with the iodophenyl group of AM251, stabilizing GPR55 in its active conformation. These differences in binding poses explain the divergent behavior of AM251 between CB1 and GPR55.

Structural comparison of LPI-bound GPR55 with the AlphaFold-predicted inactive GPR55 and antagonist YL-365-bound GPR34^14^ shows a minor outward movement of TM6 compared to other class A GPCRs (Supplementary information, Fig. S7a). However, Y288^7.53^ of the NPxxY motif in the LPI-bound GPR55 exhibits a characteristic shift toward the center of the intracellular cavity (Supplementary information, Fig. S7b), which is a hallmark of class A GPCR activation. In the DRY motif, in contrast to the polar interaction of R152^3.50^/T264^6.33^ that locks the lower part of TM6 in an inactive conformation in the antagonist-bound GPR34, R119^3.50^ of LPI-bound GPR55 forms a polar interaction with the upper residue S231^6.40^ of TM6. This interaction tilts TM6 open, allowing the αH5 of Gα to engage with the intracellular cavity of the receptor (Supplementary information, Fig. S7c).

GPR55 predominantly couples with Gα_13_.^1^ Typically, G-protein coupling is mainly mediated by the far end of αH5 of Gα. However, in the GPR55/G_13_ interaction, we also observed significant contributions from the Ras-like domain and the N-terminal helix (αN) of Gα (Fig. 1h). For the far end of αH5, receptor binding is primarily driven by two patches of hydrophobic interactions. In the first patch (patch I), L220^G.H5.16^, L224^G.H5.20^ and L229^G.H5.25^ of αH5 interact with a hydrophobic surface formed by L208^5.65^, C224^6.33^, I228^6.37^ and I123^3.54^ of GPR55 (Fig. 1h, left panel; Supplementary information, Fig. S7d). In the second patch (patch II), the third last residue of αH5, M228^G.H5.24^, inserts into a hydrophobic cavity formed by I292^8.47^, M63^2.43^, F45^1.57^, F48^1.60^ and Y56^2.36^ (Fig. 1h, left panel; Supplementary information, Fig. S7e). The insertion of the last methionine residue of αH5 into a hydrophobic cavity formed by TM1/TM2/TM7 is a unique feature of G_13_ coupling known as the ‘methionine finger pocket’.^15^ Interestingly, two polar interactions, L229^G.H5.25^/S227^6.36^ and Q226^G.H5.22^/T59^2.39^, help position these hydrophobic interactions (Fig. 1h, left panel). In contrast, the binding of the Ras-like domain to ICL3 is almost exclusively driven by extensive polar interactions. Specifically, R184^G.H4.17^ and R187^G.h4s6.09^ form salt-bridge interactions with D217^6.26^; Q190^G.h4s6.12^ forms a polar interaction with Q220^6.29^; and the backbone carbonyl group of L159^G.S4.05^ forms a hydrogen-bond interaction with T215^ICL3^ (Fig. 1h, middle panel). In the ICL2 region, K31^G.HN.51^ of αN forms a polar interaction with the backbone carbonyl group of H131^ICL2^. In addition, N223^G.H5.19^ forms hydrogen-bond interactions with P126^ICL2^ and A122^3.53^ (Fig. 1h, right panel). The extensive hydrophobic and polar interactions between Gα_13_ and GPR55 explain the exclusive coupling of G_13_ to GPR55.

GPR55 is a promising target for treating metabolic and neurological disorders, such as diabetes, chronic neurological pain, and Parkinson’s disease. Notably, the effectiveness of GPR55 agonists, such as O-1602 and AM251, have been demonstrated in animal models of neurologic and metabolic disorders. The structural information revealed by our study, particularly regarding the unique ligand recognition mode and exclusive G_13_ coupling mechanism, offers a rational basis for designing novel GPR55 modulators for associated diseases and enhances our understanding of GPR55 signaling.

## Supplementary information

Supplementary Information

Supplementary Video S1

## Data Availability

All data produced or analyzed in this study are included in the main text or the supplementary materials. The cryo-EM density maps and atomic coordinates have been deposited in the Electron Microscopy Data Bank (EMDB) and Protein Data Bank (PDB), respectively, under accession numbers: EMD-60537 and 8ZX4 for the LPI-bound GPR55/G_13_ complex; EMD-60538 and 8ZX5 for the AM251-bound GPR55/G_13_ complex.
