# Supplementary material for: Structural insight into GPR55 ligand recognition and G-protein coupling
Source: Cell Res. 2024 Oct 31;35(1):76–9. doi: 10.1038/s41422-024-01044-w (PMC11701112; doi:10.1038/s41422-024-01044-w)
Supplement: Supplementary file 1 — Supplementary Information [file 41422_2024_1044_MOESM1_ESM.pdf]

Supplementary Information

Structural insight into GPR55 ligand recognition and G-protein coupling

Ruixue Xia[1,*], Qingning Yuan[2,5*], Na Wang[1,*], Li Hou[2], Junpei Abe[3,4], Jing Song[1], Yukishige Ito[3], H. Eric Xu[2,5,6,#], Yuanzheng He[1,7,#]

[#]Corresponding: eric.xu@simm.ac.cn (H.E.X.) and ajian.he@hit.edu.cn (Y.H.)

Materials and Methods

Constructs

The codon-optimized human full-length GPR55 gene was fused with a LgBiT to its C-terminus, followed by a Tobacco etch virus (TEV) cutting site and 2× maltose-binding protein (MBP). Then the construct was cloned in pFastBac1 baculovirus expression vector. A C287V mutant was introduced by single point mutation via polymerase chain reaction (PCR). The C-terminus HiBiT fusion of human Gβ1 was cloned into pFastBac1 plasmid as the VIP1R paper[1]. The mini-Gαq and mini-$G\alpha_{13}$ constructs were adopted from the latrophilin 3/G_{13} complex paper[2], the sequences were codon-optimized and synthesized by Langjing Biotech, Shanghai, and inserted into pFastBac1. The wild-type human Gγ2 was cloned into pFastBac plasmid.

Protein expression and purification

Recombinant baculovirus encoding GPR55-LgBiT-TEV-2MBP, mini-$G\alpha_{13}$, Gβ1, and Gγ2 proteins were co-infected into Spodoptera frugiperda (Sf9) cells. The cells were cultured at 27 ℃ and 110 rpm for 48 hours, reaching a density of 2×10^6 cells per mL before infection at a ratio of 1:100 (virus volume to cell volume). Cell pellets were resuspended in a buffer containing 20 mM HEPES (pH 7.5), 150 mM NaCl, 10 mM $MgCl_2$, 20 mM KCl, 5 mM $CaCl_2$, and 0.5 mU/mL apyrase, then homogenized by douncing approximately 30 times. After incubating the lysate for 1 hour at room temperature, 0.5% (w/v) lauryl maltose neopentylglycol (LMNG, Anatrace) and 0.1% (w/v) cholesteryl hemisuccinate TRIS salt (CHS, Anatrace) were added to solubilize the membrane at 4 ℃ for 2 hours. The lysate was then ultracentrifuged at 65,000 g at 4 ℃ for 45 minutes. The supernatant was loaded onto an amylose column, incubated for 2 hours, washed with a buffer containing 20 mM HEPES (pH 7.5), 150 mM NaCl, and 0.01% LMNG/0.002% CHS, and eluted with the same buffer plus 10 mM maltose. After concentration and overnight TEV digestion at 4 ℃, the complex was injected onto a Superdex 200 Increase 10/300 GL (GE Healthcare) gel filtration column equilibrated with a buffer containing 20 mM HEPES (pH 7.5), 150 mM NaCl, 0.00075% (w/v) LMNG, 0.00025% (w/v) glyco-diosgenin (GDN, Anatrace), and 0.0002%

(w/v) CHS. The peak fractions corresponding to the GPR55/G-protein complex were concentrated to approximately 10 mg/mL and snap-frozen for later cryo-EM grid preparation. LPI or AM251 was added throughout the purification process at a concentration of 10 μM to obtain the LPI-bound or AM251-bound GPR55 complex.

Grid preparation and cryo-EM data collection

For AM251/GPR55/G_{13} complex, a 3 μL sample at 10 mg/mL was loaded to a glow-charged quantifoil R1.2/1.3 gold (Au) holey carbon grids (Quantifoil GmbH). For LPI/GPR55/G_{13} complex, a 3uL sample at 6 mg/mL was loaded to a glow-charged amorphous alloy film (M024-Au300-R12/23). Then the samples were vitrified by plunging into liquid ethane on a Vitrobot Mark IV (Thermo Fisher Scientific) instrument at setting of blot force 1, blot time 5 seconds, waiting 10 seconds, humidity 100%, temperature 4 ℃. A Titan Krios G4 equipped with a Falcon4i direct electron detector with SelectrisX and EPU were used to acquire cryo-EM movies at Advanced Center for Electron Microscopy at Shanghai Institute of Materia Medica, Chinese Academy of Sciences. The camera setting: size of 0.73 Å at a dose of 50 electron per $Å^2$ with EER mode, nominal defocus value varies from -0.8 to -1.8 μm.

Data processing

All dose-fractionated image stacks were subjected to beam-induced motion correction motion-corrected by MotionCor2[3], followed by CTF estimation via CTFFIND 4.1[4]. Particles were picked by crYOLO[5], followed reference-free 2D classification in RELION[6] (see also Supplementary Fig. S3a for details). Well-defined 2D features were used for initial model generation (cryoSPARC[7] ab initio) and 3D classification. The model was used as reference in RELION 3D classification (~5 classes). The best class with clear secondary structure features was selected for a 3D refinement in RELION, then followed to a second round 3D classification (3 classes) with mask on the complex. The selected class is subjected by a Baysian polishing[8], followed by the final refinement via the cryoSPARC Non-uniform Refinement, which generated a map of 2.85-3.03 Å, based on the gold

standard Fourier Shell Correlation (FSC) = 0.143 criterion. Local resolution estimations were performed using an implemented program in cryoSPARC.

Model building

We used AlphaFold prediction[9] of human GPR55 (AF-Q9Y2T6-F1-model_v1) was used as initial models for model rebuilding against the electron microscopy map. We used UCSF Chimera[10] to dock models into the electron microscopy density map then subjected to iterative manual adjustment in Coot[11], followed by a rosetta cryoEM refinement[12] and Phenix real space refinement[13]. Structural Figs were prepared in UCSF Chimera, ChimeraX[14] and PyMOL (https://pymol.org/2/). Ligand interaction map was drawn by LigPlus[15].

BRET assay using tricistronic activity sensors

To measure the G_{13} or G_q dissociation activity of GPR55, a tricistronic activity sensors assay was performed as previously described[16]. The G-protein sensor plasmids were obtained from Addgene (https://addgene.org/Gunnar_Schulte/). For measuring constitutive activity, 500 ng of GPCR was co-transfected with 500 ng of G protein sensor into AD293 cells in 6-well plates by Lipofectamine 2000 (Invitrogen). After incubating for 24 hours in a 37 ℃, 5% CO_2 atmosphere, the transfected cells were seeded into a poly-D-lysine hydrobromide-treated 96-well plate and incubated for another 24 hours. The cells were then washed with HBSS and incubated with a 1:1000 dilution of furimazine stock solution. The bioluminescence resonance energy transfer (BRET) ratio was measured in three consecutive readings after a 3-minute incubation at 37 ℃ using the EnVision multimode plate reader (PerkinElmer). The signal was calculated as the ratio of 460/40-nm monochromator (gain, 3600) to cpVenus emission using a 535/30-nm monochromator (gain, 4000) with an integration time of 0.3 seconds in both channels.

Measurement of receptor cell-surface expression by ELISA

GPR55 was fused with a FLAG tag on its N-terminus, followed by the HA signal peptide. To evaluate the expression levels of wild type GPR55 and mutants on cell surface, AD293 cells were transiently transfected with 500 ng plasmids encoding target receptors or vehicle plasmid (pcDNA3) using Lipofectamine 2000 (Invitrogen) reagent in a 6-well plate and incubated for 24 hours. Prior to seeding into 48-well plates, the plates were coated with a Poly-L-lysine solution at a concentration of 0.02 mg/mL for at least 30 minutes at 37 ℃. The Poly-L-lysine solution was removed by aspiration before seeding the cells. The cells were then seeded into a 48-well plate at a density of 2×10^5 cells per well and further incubated for 24 hours at 37 ℃ in 5% CO_2. The cells were subsequently fixed with 4% (w/v) paraformaldehyde for 15 minutes at room temperature and blocked with DMEM containing 10% (w/v) FBS for another hour. After washing, the cells were incubated with the mouse monoclonal anti-Flag M2 primary antibody (Sigma-Aldrich, 1:1000) at room temperature for 1 hour. Following another wash, TMB (3,3',5,5'-tetramethylbenzidine) solution was added for a color reaction, which was then stopped by adding an equal volume of 3 M H_2SO_4. The absorbance at 450 nm was determined using the EnVision multimode plate reader (PerkinElmer).

NanoBiT β-arrestin 1 recruitment assay

The C-terminus of GPR55 was fused with the Large part of NanoBiT (LgBiT) and the N-terminus of β-arrestin 1 was fused with the small part of NanoBiT (SmBiT). Similar to described before[17], AD293 cells cultured in the 6-cm culture dish were transfected with a plasmid mixture consisting of 500 ng SmBiT-β-arrestin 1, 500 ng LgBiT-fused GPR55 for one day. The transfected cells were then dispensed in 96-well plate and ligand-induced luminescent changes were measured for baseline luminescence (EnVision multimode plate reader) and 20 μL of 6X test compound (LPI 16:0, Avanti #850102) were manually added. The plate was positioned for the second measurement and luminescence counts measured from 3 min to 5 min after compound addition were normalized to the initial count. The arrestin recruitment signals were fitted to a four-parameter sigmoidal concentration-response curve (GraphPad Prism8).

Molecular dynamics simulation

The cryo-EM structure of AM251-bound GPR55 (receptor only) was used as the starting model for the MD simulation. The model was prepared in MOE and parameterized using CHARMM-GUI[18,19]. Protonation states of all titratable residues were assigned at pH 7.0. The GPR55 model was embedded in a lipid bilayer composed of POPC (palmitoyl-2-oleoyl-sn-glycero-3-phosphocholine) and cholesterol in a 4:1 ratio. The membrane dimensions were 65 x 65 Å, with 22.5 Å of water above and below, resulting in overall system dimensions of approximately 65 x 65 x 120 Å. Ion concentration was set to 0.15 M KCl. The Amber force fields were applied as follows: FF19SB for the protein, LIPID17 for the lipid, TIP3P for water, and GAFF2 for the ligand. Simulations were run using the Amber20 package[20]. The system underwent initial energy minimization for the solvent and all atoms, followed by heating to 300 K over 300 ps and equilibration for 700 ps. Three independent production runs of 200 ns each were then conducted with a 2 fs time step. Long-range electrostatic interactions were calculated using the Particle Mesh Ewald algorithm, and a 10 Å cutoff was used for short-range electrostatic and van der Waals interactions. The SHAKE algorithm was applied to constrain all bonds involving hydrogen atoms. Temperature (300 K) and pressure (1 atm) were maintained using a Langevin thermostat and Berendsen barostat, respectively. Trajectory analysis and visualization were performed using VMD[21], with video recording also handled through VMD.

Supplementary video title

Supplementary information, Video S1 Movie track of MD simulation of AM251-bound

GPR55. The time length is 200 ns, interval is 5 steps.

References

1. Duan, J. *et al.* Cryo-EM structure of an activated VIP1 receptor-G protein complex revealed by a NanoBiT tethering strategy. *Nature communications* **11**, 4121, doi:10.1038/s41467-020-17933-8 (2020).
2. Barros-Alvarez, X. *et al.* The tethered peptide activation mechanism of adhesion GPCRs. *Nature*, doi:10.1038/s41586-022-04575-7 (2022).
3. Zheng, S. Q. *et al.* MotionCor2: anisotropic correction of beam-induced motion for improved cryo-electron microscopy. *Nature methods* **14**, 331-332, doi:10.1038/nmeth.4193 (2017).
4. Rohou, A. & Grigorieff, N. CTFFIND4: Fast and accurate defocus estimation from electron micrographs. *Journal of structural biology* **192**, 216-221, doi:10.1016/j.jsb.2015.08.008 (2015).
5. Wagner, T. *et al.* SPHIRE-crYOLO is a fast and accurate fully automated particle picker for cryo-EM. *Commun Biol* **2**, 218, doi:10.1038/s42003-019-0437-z (2019).
6. Fernandez-Leiro, R. & Scheres, S. H. W. A pipeline approach to single-particle processing in RELION. *Acta Crystallogr D Struct Biol* **73**, 496-502, doi:10.1107/S2059798316019276 (2017).
7. Punjani, A., Rubinstein, J. L., Fleet, D. J. & Brubaker, M. A. cryoSPARC: algorithms for rapid unsupervised cryo-EM structure determination. *Nature methods* **14**, 290-296, doi:10.1038/nmeth.4169 (2017).
8. Zivanov, J., Nakane, T. & Scheres, S. H. W. A Bayesian approach to beam-induced motion correction in cryo-EM single-particle analysis. *IUCrJ* **6**, 5-17, doi:10.1107/S205225251801463X (2019).
9. Jumper, J. *et al.* Highly accurate protein structure prediction with AlphaFold. *Nature* **596**, 583-589, doi:10.1038/s41586-021-03819-2 (2021).
10. Pettersen, E. F. *et al.* UCSF Chimera--a visualization system for exploratory research and analysis. *J Comput Chem* **25**, 1605-1612, doi:10.1002/jcc.20084 (2004).
11. Emsley, P. & Cowtan, K. Coot: model-building tools for molecular graphics. *Acta Crystallogr D Biol Crystallogr* **60**, 2126-2132, doi:10.1107/S0907444904019158 (2004).
12. Wang, R. Y. *et al.* Automated structure refinement of macromolecular assemblies from cryo-EM maps using Rosetta. *eLife* **5**, doi:10.7554/eLife.17219 (2016).
13. Adams, P. D. *et al.* PHENIX: a comprehensive Python-based system for macromolecular structure solution. *Acta Crystallogr D Biol Crystallogr* **66**, 213-221, doi:10.1107/S0907444909052925 (2010).
14. Pettersen, E. F. *et al.* UCSF ChimeraX: Structure visualization for researchers, educators, and developers. *Protein science : a publication of the Protein Society* **30**, 70-82, doi:10.1002/pro.3943 (2021).
15. Wallace, A. C., Laskowski, R. A. & Thornton, J. M. LIGPLOT: a program to generate schematic diagrams of protein-ligand interactions. *Protein engineering* **8**, 127-134, doi:10.1093/protein/8.2.127 (1995).

16 Schihada, H., Shekhani, R. & Schulte, G. Quantitative assessment of constitutive G protein-coupled receptor activity with BRET-based G protein biosensors. *Sci Signal* **14**, eabf1653, doi:10.1126/scisignal.abf1653 (2021).

17 Xu, Z. *et al.* Structural basis of sphingosine-1-phosphate receptor 1 activation and biased agonism. *Nat Chem Biol*, doi:10.1038/s41589-021-00930-3 (2021).

18 Wu, E. L. *et al.* CHARMM-GUI Membrane Builder toward realistic biological membrane simulations. *J Comput Chem* **35**, 1997-2004, doi:10.1002/jcc.23702 (2014).

19 Lee, J. *et al.* CHARMM-GUI Input Generator for NAMD, GROMACS, AMBER, OpenMM, and CHARMM/OpenMM Simulations Using the CHARMM36 Additive Force Field. *J Chem Theory Comput* **12**, 405-413, doi:10.1021/acs.jctc.5b00935 (2016).

20 Case, D. A. *et al.* The Amber biomolecular simulation programs. *J Comput Chem* **26**, 1668-1688, doi:10.1002/jcc.20290 (2005).

21 Humphrey, W., Dalke, A. & Schulten, K. VMD: visual molecular dynamics. *J Mol Graph* **14**, 33-38, 27-38, doi:10.1016/0263-7855(96)00018-5 (1996).

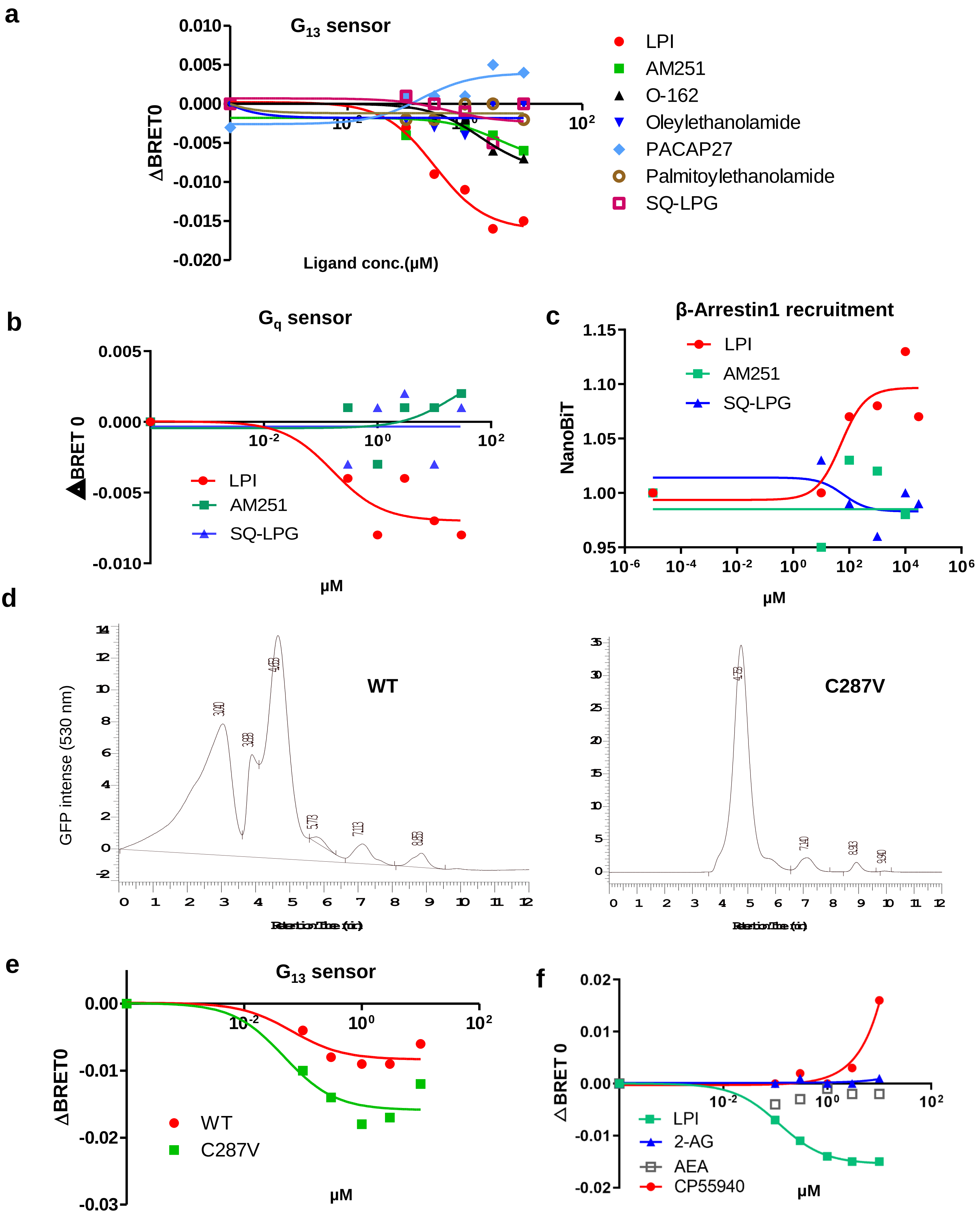

Supplementary information, Fig. S1 Characterization of GPR55. a, Examination of various GPR55 ligands in a BRET G_{13} dissociation assay. **b**, Examination of GPR55 ligands in a BRET G_q dissociation assay. **c**, Examination of various GPR55 ligands in a NanoBiT β-arrestin recruitment assay. **d**, FSEC profile of WT and C287V mutant of GPR55. **e**, Examination of WT and C287V activities in a BRET G_{13} dissociation assay. **f**, BRET G13 dissociation assay of endogenous or synthetic cannabinoids. **a-c** and **e-f**, Data are presented as mean values ± SD; *n* = 3.

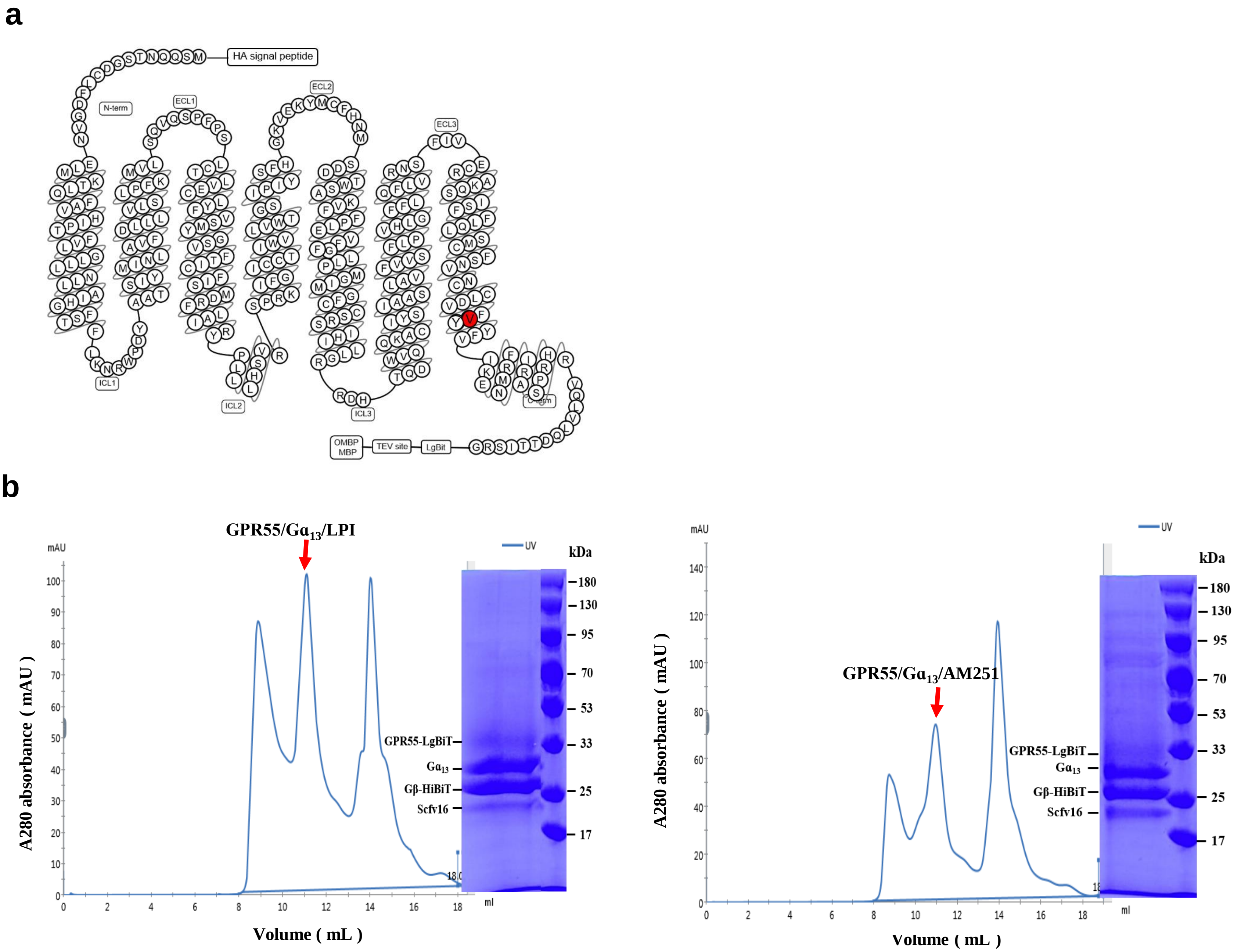

Supplementary information, Fig. S2 Expression and purification of GPR55. a, A snake-shape diagram of the GPR55 construct used in complex assembling, the diagram was adopted from GPCRdb. **b**, Size exclusion column profiles and SDS-PAGE analysis of GPR55/G_{13} complexes.

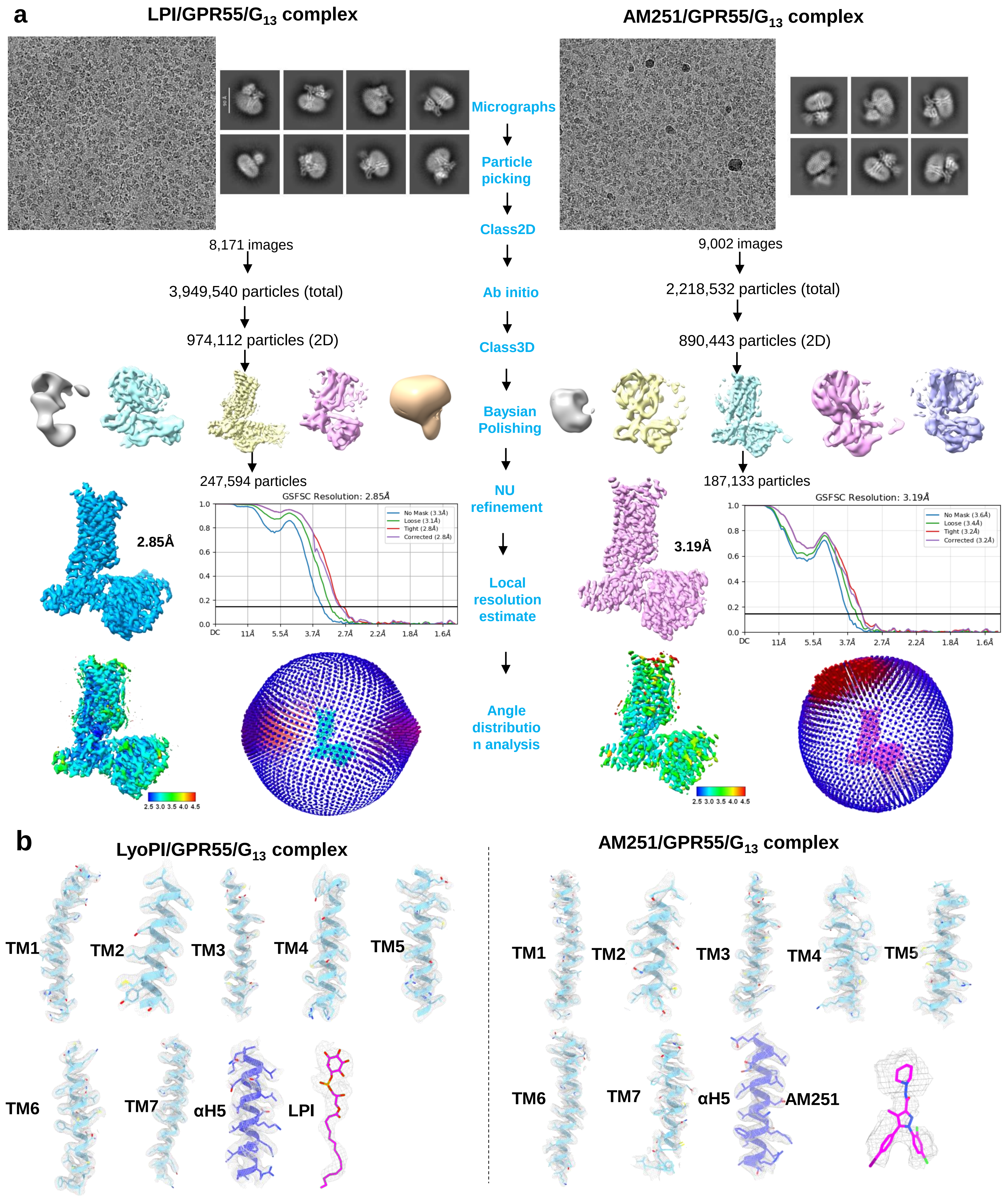

Supplementary information, Fig. S3 Single particle analysis of GPR55/G_{13} complexes. a, Flow-chart of cryo-EM data process of GPR55/G_{13} complexes. **b**, Cryo-EM density map of representative regions of GPR55/G_{13} complexes.

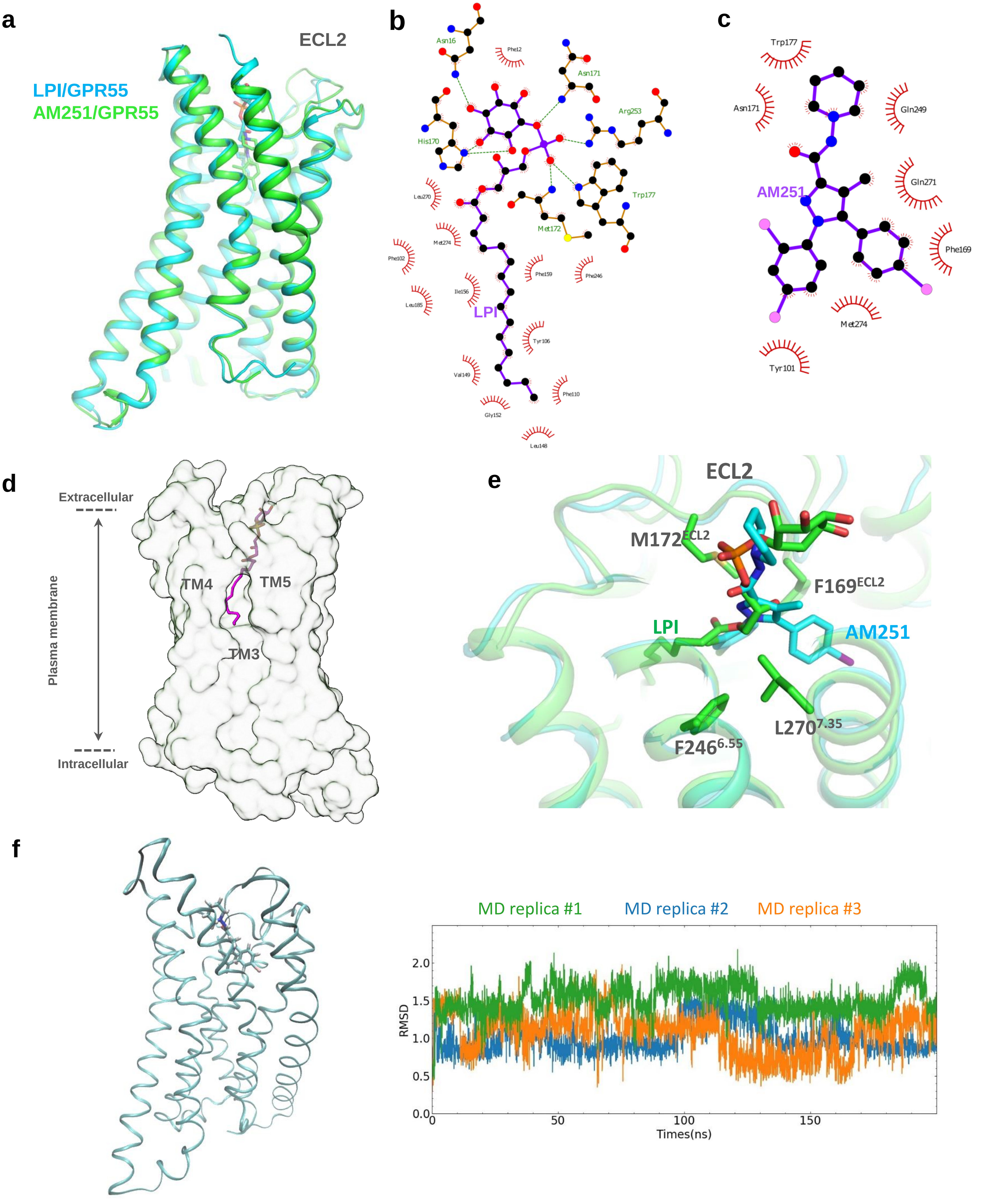

Supplementary information, Fig. S4 Additional information of ligand binding pocket of GPR55. a, A comparison of LPI-bound GPR55 with AM251-bound GPR55. **b**, A schematic LPI/GPR55 interaction map drawn by LigPlus. **c**, A schematic AM251/GPR55 interaction map drawn by LigPlus. **d**, The lateral entry of GPR55 for lipid ligands from the plasma membrane. **e**, A superimposition of AM251 binding pose with LPI binding pose in GPR55. **f**, MD simulations of AM251-bound GPR55. Left panel, a snapshot of AM251 binding in the MD simulations; right panel, trajectory analysis of AM251 in the simulations.

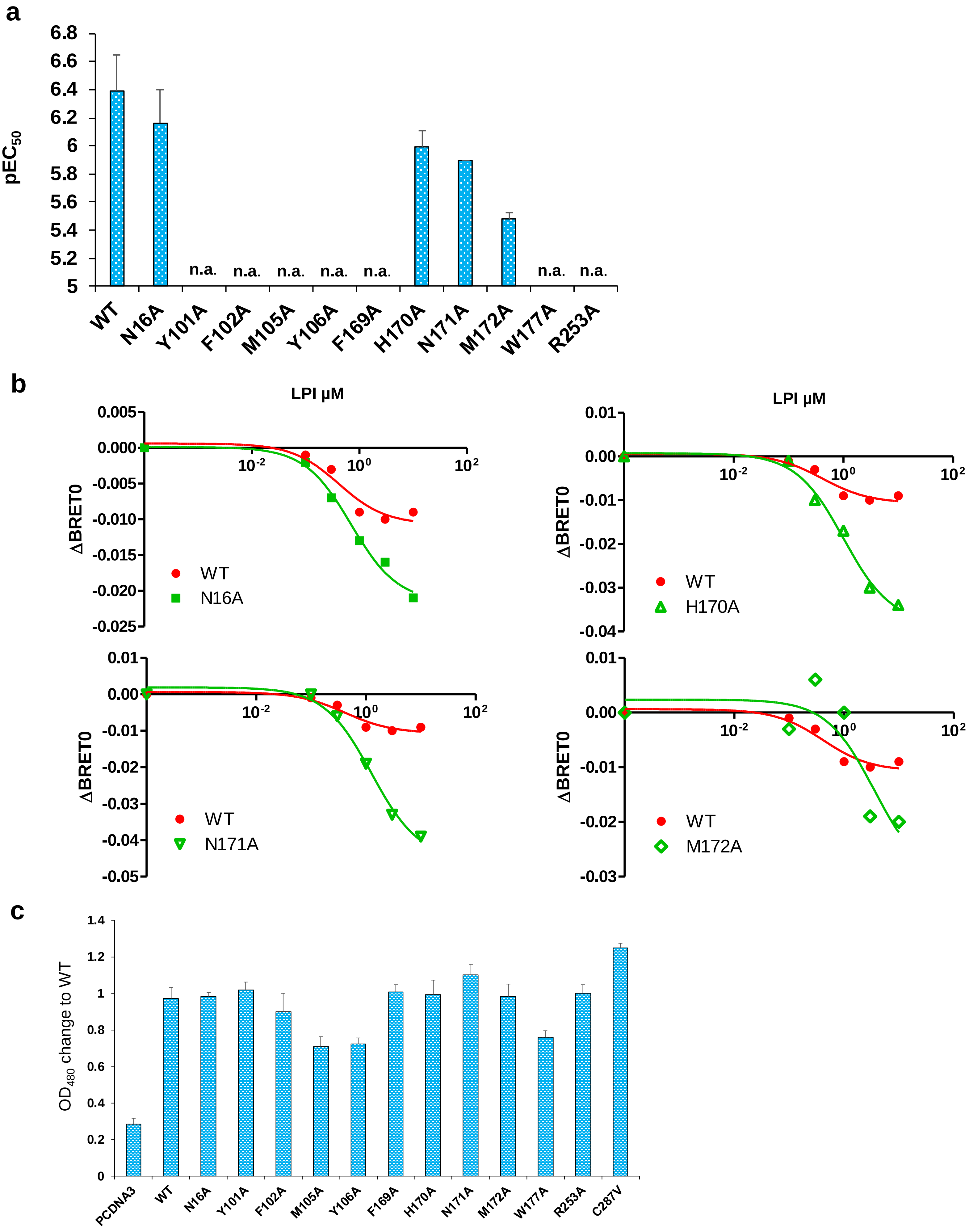

Supplementary information, Fig. S5 Additional information of GPR55 ligand recognition. a, A plot of pEC_{50} of LPI for different GPR55 mutants. Data are presented as mean values ± SD; n = 3 independent samples. n.a., data not available due to poor curve fit. **b**, Dose response of GPR55 pocket mutants to LPI in a BRET G_{13} dissociation assay. Data are presented as mean values ± SD; n = 3 independent samples. **c**, Surface expression of GPR55 mutants. Data are presented as mean values ± SD; n = 3 independent samples.

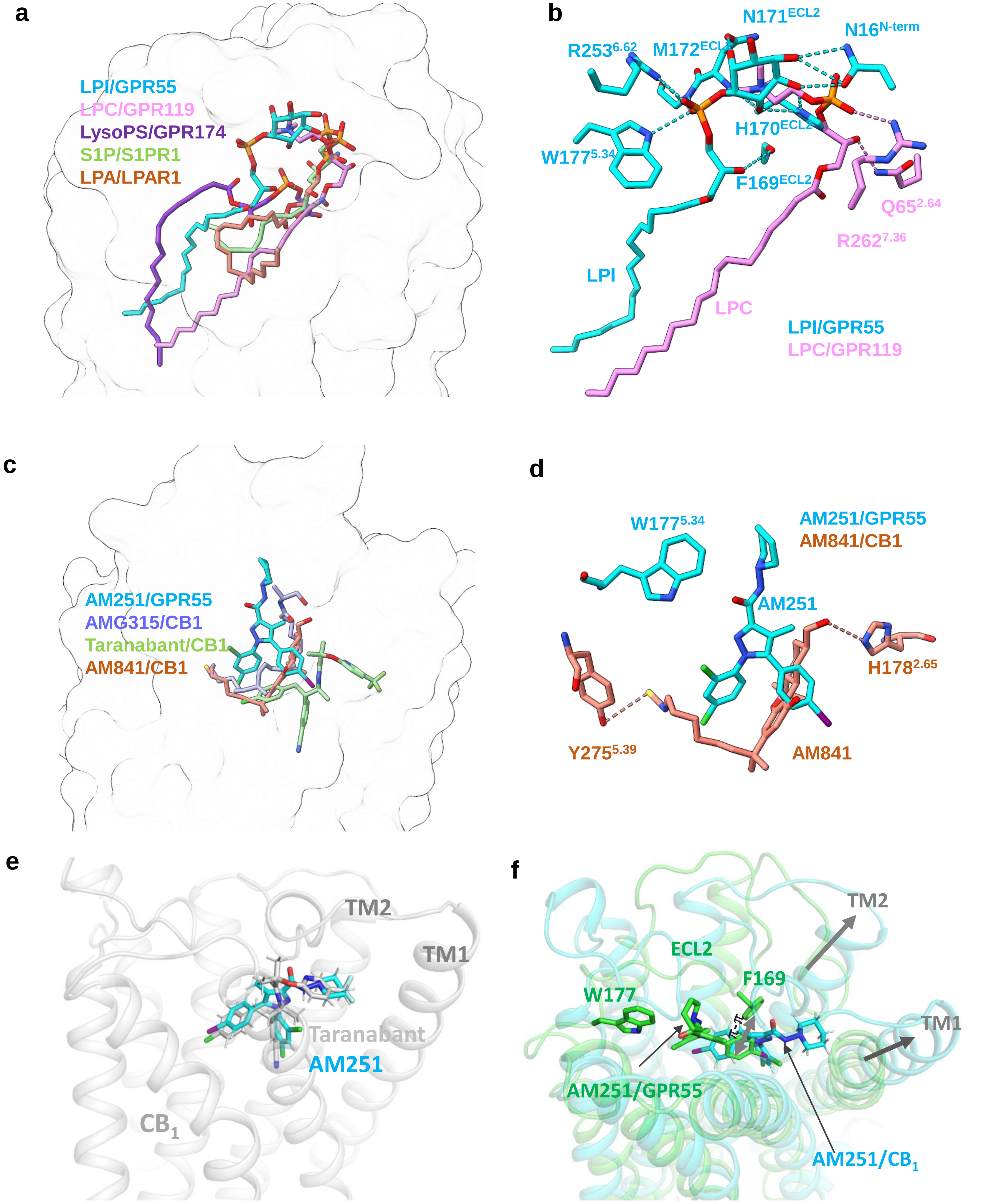

Supplementary information, Fig. S6 Comparisons of GPR55 ligand binding modes. **a**, A comparison of LPI binding mode with other lysophospholipids binding modes. LPC/GPR119 (PDB:7xz5), LysoPS/GPR174 (PDB:7xv3), S1P/S1PR1 (PDB:7wf7), LPA/LPAR1 (PDB:7td0). **b**, A detailed comparison of LPI binding mode in GPR55 with LPC binding mode in GPR119. **c**, A comparison of AM251 binding mode in GPR55 with cannabinoid analogs binding modes in CB_1. AM841/CB_1 (PDB:6kpg), AMG315/CB_1 (PDB:8ghv), taranabant/CB_1 (PDB:5u09). **d**, A detailed comparison of AM251 binding mode in GPR55 with AM841 binding mode in CB_1. **e**, Docking of AM251 in the taranabant-bound CB_1 (PDB:5u09). **f**, A superimposition of AM251-bound GPR55 with the AM251-docked CB_1.

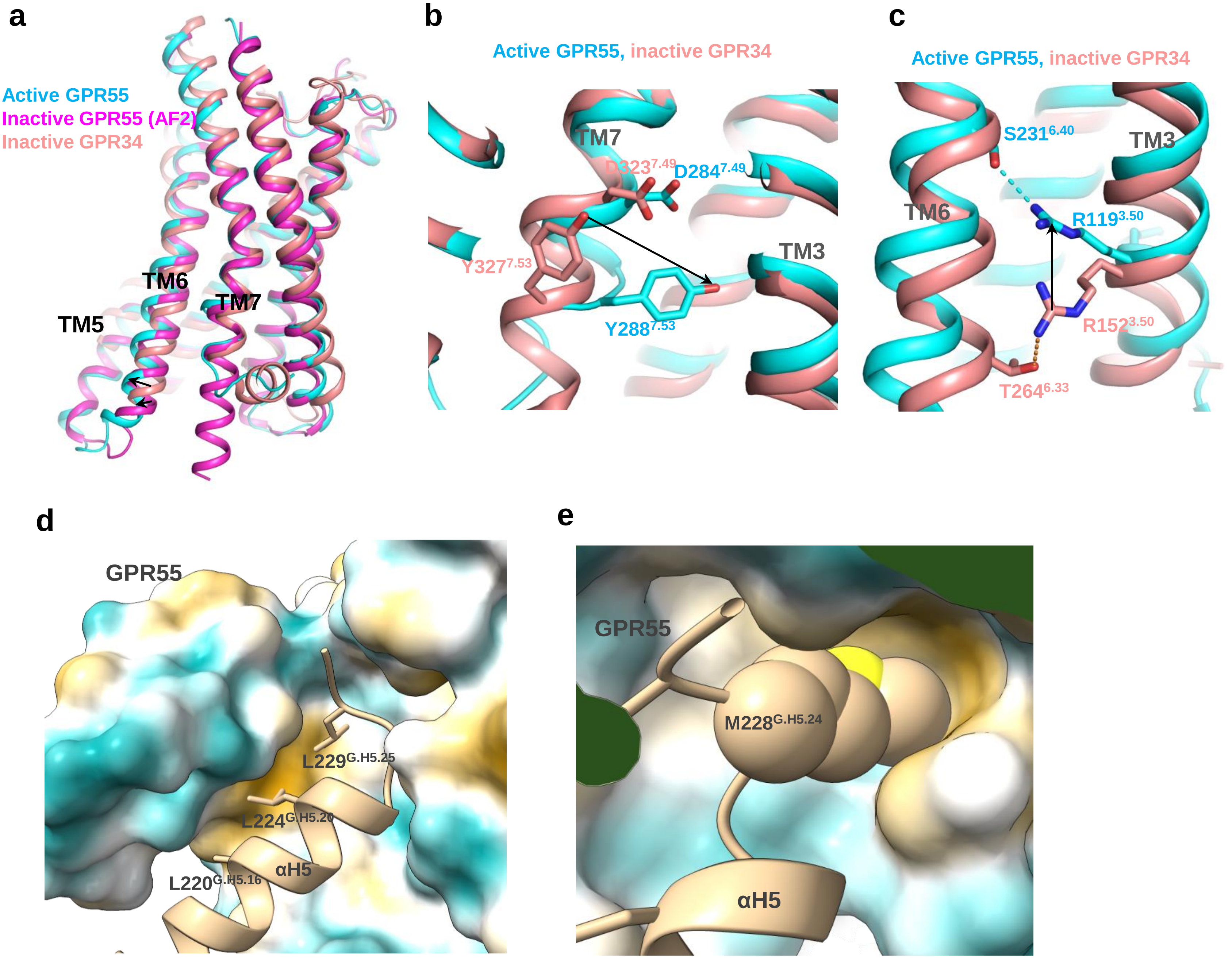

Supplementary information, Fig. S7 GPR55 activation and G_{13} coupling. **a**, A comparison of LPI-bound GPR55 (active) with AlphaFold 2 (AF2) prediction of inactive GPR55 and antagonist YL-365-bound inactive GPR34 (PDB:8iyx). **b**, Conformation change of the NPxxY motif in GPR55 activation. **c**, Conformation change of the DRY motif in GPR55 activation. **d**, A surface hydrophobicity analysis on GPR55 for the patch I hydrophobic interaction between αH5 and the receptor. **e**, A surface hydrophobicity analysis of the intracellular cavity in GPR55 that M228 inserts into.

Supplementary information, Table S1. Activity of GPR55 ligands in BRET G_{13} dissociated assay

	LPI	AM251	O-162	Oleylethanolamide	PACAP27	palmitoyletholanine	SQ-LPG
pEC50	6.514	5.4064	5.7277	NA	NA	NA	6.2868
SD (+/-)	0.01636	0.005752	0.008644	NA	NA	NA	0.003087
Emax	-0.016	-0.006	-0.007	NA	NA	NA	-0.005
SD (+/-)	0.0007306	0.00212	0.0006885	NA	NA	NA	0.001211
n	3	3	3	3	3	3	3

Supplementary information, Table S2. Cryo-EM data collection and refinement statistics

	LPI/GPR55/miniG$_{13}$<br>EMD-60537<br>8ZX4	AM251/GPR55/miniG$_{13}$<br>EMD-60538<br>8ZX5
**Data collection and processing**		
Magnification	165,000	165,000
Voltage (kV)	300	300
Electron exposure (e^-/Å^2)	50	50
Defocus range (μm)	0.8-1.8	0.8-1.8
Pixel size (Å)	0.73	0.73
Symmetry imposed	C1	C1
Initial particle image (no.)	3,949,540	2,218,532
Final particle image (no.)	247,594	187,133
Map resolution (Å)	2.85	3.19
FSC threshold	0.143	0.143
**Refinement**		
Initial model used (PDB code)	AF-Q9Y2T6-F1-model_v1	AF-Q9Y2T6-F1-model_v1
Model Resolution (Å)	NA	NA
Map sharpening *B* factor (Å^2)	-121.5	-156
Model composition		
Non-hydrogen atoms	6753	6106
Protein residues	883	821
Ligands	1	1
B factor (Å^2)		
Protein	61.19	59.59
Ligand	76.7	74.32
R.m.s. deviations		
Bond length (Å)	0.003	0.006
Bond angles (°)	0.643	1.149
Validation		
MolProbity score	1.79	1.68
Clashscore	9.24	5.19
Poor rotamers (%)	0	0
Ramachandran plot		
Favored (%)	95.64	93.94
Allowed (%)	4.36	6.06
Disallowed	0	0

Supplementary information, Table S3. Activity of GPR55 mutants in BRET G_{13} dissociated assay by LPI

	WT	N16A	Y101A	F102A	M105A	Y106A	F169A	H170A	N171A	M172A	W177A	R253A
pEC_{50}	6.3942	6.161263	NA	NA	NA	NA	NA	5.99218	5.894	5.476067	NA	NA
SD (+/-)	0.252347	0.234858	NA	NA	NA	NA	NA	0.118458	0.003122	0.050404	NA	NA
Emax	-0.011	-0.016	NA	NA	NA	NA	NA	-0.034	-0.036	-0.02	NA	NA
SD (+/-)	0.0008	0.001	NA	NA	NA	NA	NA	0.0012	0.0013	0.001	NA	NA
n	3	3	3	3	3	3	3	3	3	3	3	3
